# Chitin from the Mollusc Chiton: Extraction, Characterization and Chitosan Preparation

**Published:** 2017

**Authors:** Hashem Rasti, Kazem Parivar, Javad Baharara, Mehrdad Iranshahi, Farideh Namvar

**Affiliations:** a *PhD student, Department of Biology, Science and Research Branch, Islamic Azad University, Tehran, Iran.*; b *Professor,Department of Biology, Science and Research Branch, Islamic Azad University, Tehran, Iran. *; c *Professor, Research Center for Animal Development, Applied Biology & Biology Department, Mashhad Branch, Islamic Azad University, Mashhad, Iran. *; d *Professor, Biotechnology Research Center, School of Pharmacy, Mashhad University of Medical Sciences, Mashhad, Iran. *; e *Assistant professor, Institute of Tropical Forestry and Forest Products (INTROP), University Putra Malaysia,UPM Serdang, Selangor 43400, Malaysia.*

**Keywords:** Chitin, Chiton, Chitosan, Persian Gulf, Antioxidant, Natural resource

## Abstract

This study presents the first ever data of extracting chitin from the Chiton shell, which was then converted to the soluble chitosan by soaking in the 45% NaOH solution. The obtained chitin and chitosan were characterized by the seven different methods. Antioxidant activity of the extracted chitosan was also evaluated using the two methods. The shell content was divided into calcium carbonate (90.5 %), protein (5.2%), and chitin (4.3 %). Due to the results of element analysis and ^1^H NMR, the final degree of deacetylation of chitosan was 90%. Surprisingly, a significant amount of Fe was accidentally found in the shell after demineralization, and removed from the solution through the filtering. Nonetheless, remained Fe in the extracted chitin and chitosan was 20 times higher than those previously reported from the shell of shrimps and crabs. Presence of this amount of Fe could describe why the produced chitosan was darker compared to the commercial chitosan. Antioxidant activity tests showed that the IC_50_ of the extracted chitosan was higher than one estimated for the commercial chitosan. Antioxidant activity of the extracted chitosan is even better than the commercial version and may be used in pharmaceutical industry as a source of antioxidant.

## Introduction

Natural products are diverse secondary bioactive metabolites with significant roles in regulating various biological systems. Marine environment is wealthy of unbeatable effective natural products. Due to the specific characteristics of the marine environment including physical and chemical conditions, marine organisms consist bioactive molecules with unbeatable properties (1). In recent years, oceans have been considered as a source of sufficient natural products (2).

Molluscs in the taxon polyplacophora, commonly named Chiton, are known as the live fossils since their body structure has not significantly changed for over 300 million years (3). This taxon is comprised of more than 940 live species and about 430 fossil species (4, 5). The shell of these animals includes eight aragonite segments and is oval in shape with a size ranged from a few millimetres to 15 centimetres. The meaty part of their body is used in food industry (6). 

Chitin, the second most common polysaccharide on the planet after the cellulose, is a none elastic and nitrogenous natural polymer structured as a linear chain by the 2-acetoamido-2- deoxy-β-D-glucopyranose monomers (7, 8). In nature, chitin presences as the α-, β-, and γ-forms, are usually extractable from the exoskeleton of crustaceans, squid pens, and fungi, respectively (9,10). The extraction of chitin comprised two stages; demineralization using HCl to remove calcium carbonate, and deproteinization using a NaOH solution to remove protein. 

Chitosan, a linear polysaccharide consisted of (1-4)-linked 2-amino-2-deoxy-b-D-glucopyranose monomers, is made by deacetylation of the extracted chitin. The chitin deacetylation using strong alkaline medium has been considered as the main method for providing chitosan (11, 12). Whenever the degree of deacetylation (DDA) reaches higher than approximately 90%, chitosan becomes soluble in acidic solutions (13, 14). 

Chitin and its derivatives, mainly chitosan, have useful biological properties, such as being biocompatible and biodegradable, and exhibit antimicrobial activity, wound healing properties, and haemostatic activity (15, 16 and 17). The extraction of β chitin has been done from *Sepia pharaonis *sp. cuttlebone in Persian Gulf (18). It has also been extracted from exoskeleton of blue swimming crab in Persian Gulf successfully (19). In addition, chitin extraction and producing chitosan from brine shrimp (Artemia urmiana) has been done (20). 

There are many applications introduced for chitin and chitosan; both are useable for example in cosmetics, agriculture, food industry, biomedicine, pharmacy, paper industry and wastewater purification (21, 22). The main goal of this study was to access the possibility of extracting chitin from the shell of the Persian Gulf Chiton collected from the rocky shore lying in the southern Qeshm Island, northeastern Persian Gulf. The results of this study may be useful to introduce novel natural resources of chitin for using in medical and pharmaceutical researches.

## Materials and methods

*Sample collection and preparation*

**Scheme 1:**
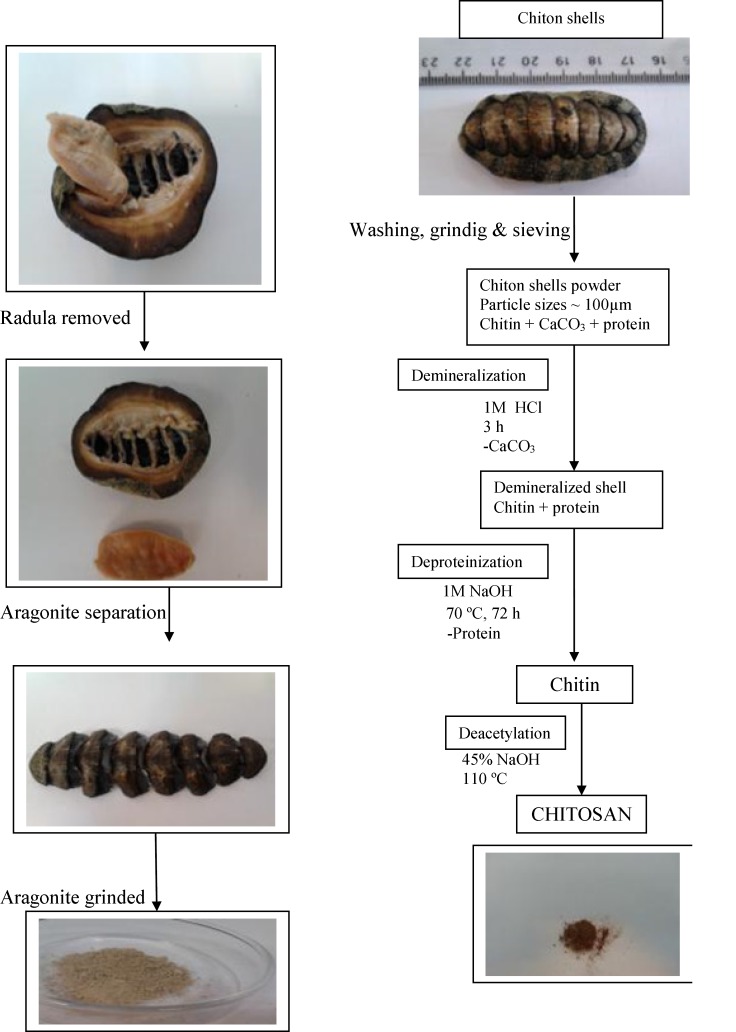
Extraction of chitin and preparation of chitosan

The Chitons were collected from rocky intertidal habitat lying at the southern Qeshm Island, north-eastern Persian Gulf. Samples were placed in ice box and shipped to the laboratory as soon as possible. In the laboratory, meaty parts of the molluscs were removed, and then remained hard shells were washed with water and dried in oven over night at 70 ºC. The dried shells (aragonite) were weighed and then powdered using a mixer mill 400 mm machine (scheme 1). 

*Three steps for the chitosan production*

*Demineralization*

Demineralization was carried out at the room temperature using 1 M hydrochloric acid solution (40 millilitre per gram) for 3 h. The shell powder was slowly added to the acid. Combination of these two substances made carbon dioxide bubbles. The resulting precipitant was washed using distilled water. Then, the demineralized samples were dried at 70 ºC for 24 h and weighed by a lab scale (23).

*Deproteinization*

Deproteinization was carried out using 1 M sodium hydroxide solution (20 millilitre per gram) at 70 ºC. The deproteinization process lasted three days. The colour of the medium had been become dark during the first 24 h, thus the medium was changed every 2 h and fresh sodium hydroxide was added. After two days, the colour gradually changed. The dark colour was not observed after the end of the third and the solution became to be finally clear, which was supposed as an index of full deproteinization. The resulting precipitant was washed with distilled water, and then with hot ethanol (10 millilitre per gram) for 3 h. After washing, the precipitant was boiled in acetone for 1 h to remove any impurities. The final resulting precipitant was dried at 70 ºC for 24 h and weighed by a lab scale (23). 

*Deacetylation*

This process was carried out by solving the deproteinized product in 45% sodium hydroxide solution (15 millilitre per gram) at 110 ºC for 5, 15 and 24 h. The heating time has been enhanced in the order of increasing degree of deacetylation. The product gained after 24 hours heating was soluble in 2% acetic acid, indicating a high degree of deacetylation. Then, the resulting product was continuously washed with distilled water and filtered in order to separate the solid matter, which was supposed as the final product (23). 

*Characterization methods*

*Fourier transform infrared spectroscopy (FTIR)*

IR spectra were measured by a KBr-supported sample of chitin and chitosan over the frequency range 4000–400 cm^-1^ at a resolution of 4 cm^-1^ using a model of 2000 Perkins-Elmer spectrometer. 

The sample was thoroughly mixed with KBr; the dried mixture was then pressed to result in a homogeneous sample/KBr disc (24).

*X-ray powder diffractometry (XRD)*

X-ray diffraction analysis (XRD) was applied to detect the crystallinity of the extracted samples of chitin and their corresponding chitosan. A Scintag powder diffractometer was used for this purpose between 2θ angles of 5^0^and 40^0^; Ni-filtered Cu Ka-radiation was used as the X-ray source. The relative crystallinity of the polymers was calculated by dividing the area of the crystalline peaks into the total area under the curve (25).

*Elemental analysis*

A Costech CHNS-4010 elemental analysis apparatus was used to determine the amount of C and N in the chitosan (26). In this case, the samples were heated to a temperature of 1000 ºC and approximately 2 mg of the product was placed inside a silver capsule and dropped into the CHNS-4010 furnace, where it was completely combusted. This instrument relies upon infra-red detection to measure the weight percentage of carbon, while nitrogen was measured by thermal conductivity detection. The DDA of chitosan was determined by the formula as follows:

DDA %=6.857-CN1.7143×100           (27)

*Atomic absorption*

The powder was weighed before acidic digestion and performance of the final preparation step. One gram of the powder was digested in HNO_3_. The concentrations of sodium, potassium, calcium, magnesium, and iron were determined in the digested solution using the atomic absorption machine (Model: AA360).

*Nuclear magnetic resonance (NMR)*

NMR spectra were recorded using a Bruker Avance 400 spectrometer in 2% deuterated trifluoroacetic acid (TFA) in D_2_O solution. The process was run at 70 ºC, which the solvent (HOD) peak did not interfere with any chitin and chitosan peaks. After dissolution, approximately 1 mL of the chitin and deacetylated powder digested in acid solutions were transferred to a 5 mm NMR tube. The sample tubes were inserted in the magnet and allowed to reach thermal equilibrium for 10 min before performing process (24). 

*Scanning electron microscopy (SEM)*

Scanning electron microscopy (SEM) was used for inspecting the topographies of the samples at ambient magnifications. The samples were gold coated under nitrogen atmosphere using a Bal-Tec SCD 005 sputter coater and the surface morphology was recorded at room temperature using a Scanning Electron Microscope, Philips XL30 with an acceleration voltage of 20 kV (28).

*Energy dispersive X-ray spectroscopy (EDX)*

Energy dispersive X-ray spectroscope (DX-700HS Shimadzu, Japan) was used to identify the elemental composition of the samples. The apparatus was connected to the SEM to allow for elemental data to be collected about the specimen under examination.

*Two tests used to evaluate antioxidant activity*

Two common tests were used to evaluate antioxidant activity of chitosan. 

*DPPH (2,2-diphenyl-1-picrylhydrazyl) radical scavenging assay*

Free radical scavenging activity of extracted chitosan in comparison to commercial chitosan was measured by using its strength to snare the DPPH free radicals. For this target, DPPH working solutions were prepared by dissolving 1 mg DPPH in 10 mL ethanol. Firstly, extracted chitosan and commercial chitosan dissolved in acetic acid glacial 2% and then various concentration of chitosan was prepared by serial dilution method. The reaction mixture contained 1 mL DPPH working solution and 1 mL chitosan in various concentrations. After 30 min incubation at room temperature absorbance of sample was read at 517 nm. Butylated hydroxyl anisole (BHA) was used as a standard compound. The discoloration activity was calculated using the following equation (1). 

$\mathrm{DPPH radical scavenging}(\%)=100\times \left(\frac{{\mathrm{absorbance.}}_{\mathrm{control group}}-{\mathrm{absorbance.}}_{\mathrm{sample group}}}{{\mathrm{absorbance.}}_{\mathrm{control group}}}\right)$

*ABTS (2,2′-azinobis-3-ethylbenzothiazoline-6-sulfonic acid) radical scavenging assay *

 ABTS radical scavenging activity of the chitosan was determined according to the method of Soltani *et al*. (2014). Briefly, the ABTS^+^ solution was constructed by the reaction of 7 mM of ABTS solution in 2.45 mM potassium per sulfate (final concentration). The blend was kept in the dark at room temperature for 12-16 hours before use. The ABTS^+^ working solution was prepared by dilution of the ABTS^+^ stock solution and distilled water to increase a 0.70±0.02 absorbance at 734 nm. The reaction blend was prepared by mixing 1 mL of the working solution in 1 mL of various concentrations of chitosan. After incubation for 1 hour at room temperature in dark, absorbance was taken at 734 nm (1).

## Results

*Chemical composition of Chiton shells*

**Figure 1:**
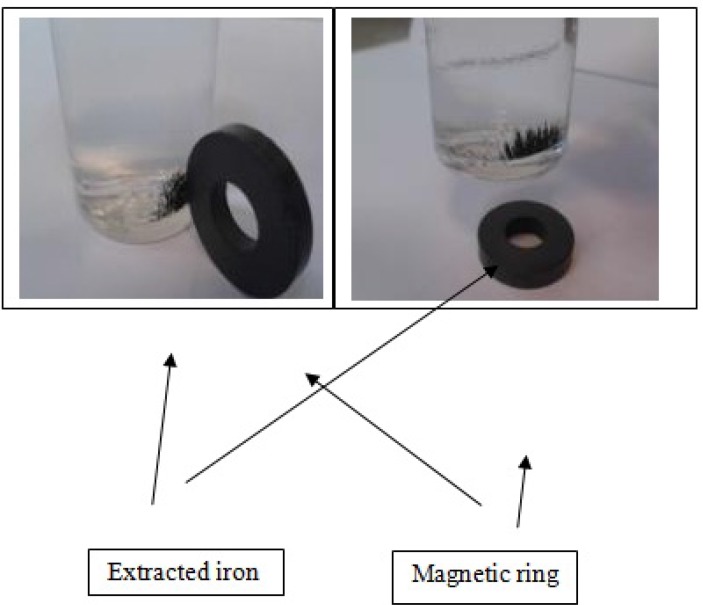
Iron extracted from the Chiton shells after demineralization

Chiton shell powder ﴾65g﴿ was used for the extraction process. The analysing of the components through demineralization and deproteinization showed that the Chiton shells comprised of 90.5% calcium carbonate, 5.2% protein and 4.3% chitin. Surprisingly, a significant amount of iron (Fe) was observed at the bottom of bottle after filtering process in demineralization, which was provided by moving toward the magnet (Figure 1). Based on the results obtained from the atomic absorption method in our study, the concentrations of Fe in deacetylated product was 47.2 ppm.

*Chitin and chitosan characterization*

*FTIR analysis*

**Figure 2:**
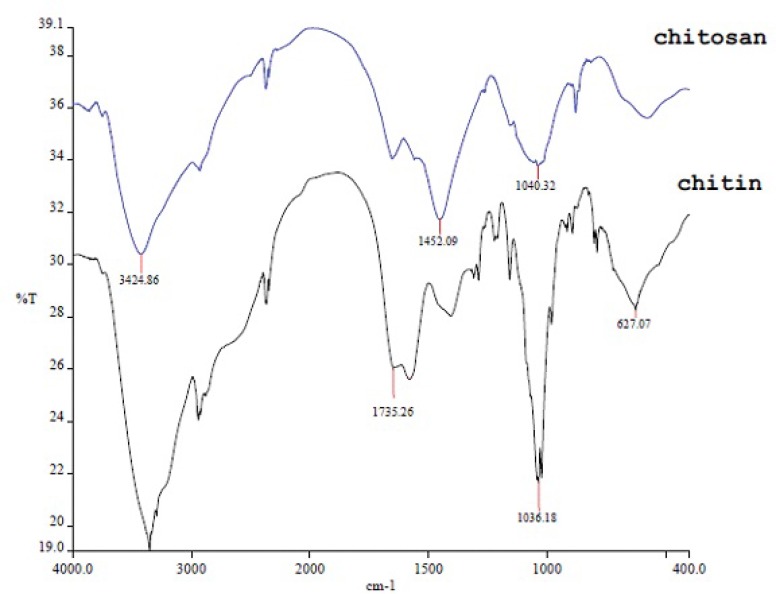
FT-IR spectra of chitin and chitosan

The seven characterization tests used in this study showed that the deproteinized and deacetylated products were chitin and chitosan, respectively. In detail, the results of fourier transform infra-red spectroscopy, used to determine the activated groups of chemical components, is given in Figure 2. According to this spectrum, the chitin sharp peaks were observed at 627.07 cm^-1^ (out of plane OH), 1036.18 (C-O), 1735.26 (C=O), 3200-3500 (N-H, O-H) (Figure 2). Further, the chitosan sharp peaks were observed at 1040.32 cm^-1^ (C-O), 1452.09 (CH_2_ in the CH_2_OH group), and 3424.86 (NH in secondary amides and NH_2_ in primary amines) (Figure 2). 

*Elemental analysis*

The influence of heating time in 45% NaOH solution is showed in Table 1. The value of DDA% calculated as 31%, 52%, and 91% after 5, 15, and 24 h of heating, respectively. These results showed that the heating time has a positive effect on the degree of deacetylation.

*X-ray powder diffractometry (XRD)*

**Figure 3:**
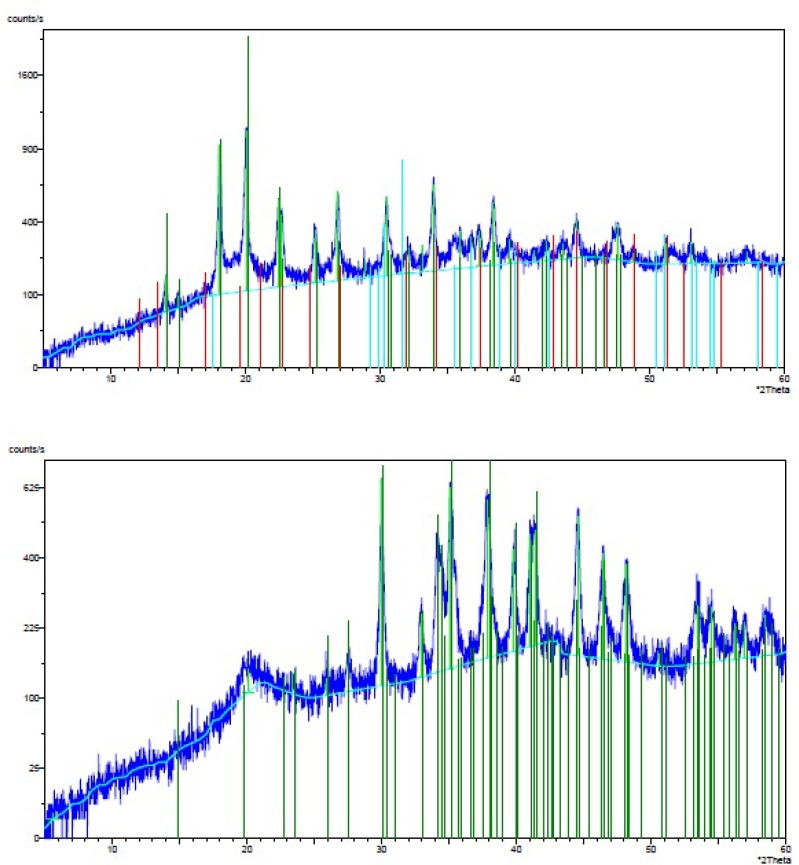
XRD of (A) chitin and (B) its corresponding chitosan

X-ray diffraction (XRD) analysis was applied to detect the crystallinity of the isolated chitin and the obtained chitosan. The XRD pattern of chitin around 5-60° showed eleven sharp crystalline reflections, whereas the stronger sharp reflections were observed around 15-35° (18°, 20°, 23°, 25°, 27°, 30°, and 34°), with the strongest sharp reflection at 2θ around 18-20° (1100° count/s). Further, strongest sharp reflection for chitosan was observed at 2θ around 30-35° (625° count/s) (Figure 3). Therefore, the XRD pattern of chitosan showed that the crystallinity of chitosan was reduced compared to chitin, because the peaks of chitosan were shifted to higher 2θ-20°. 

*Atomic absorption*

Atomic absorption assay was used to investigate the presence and concentrations of five target elements in the extracted chitosan; the determined concentrations of sodium, potassium, calcium, magnesium, and iron were 194.93, 0.093, 0.310, 0.162 and 47.200 ppm, respectively. 

*Nuclear magnetic resonance (NMR)*

**Figure 4:**
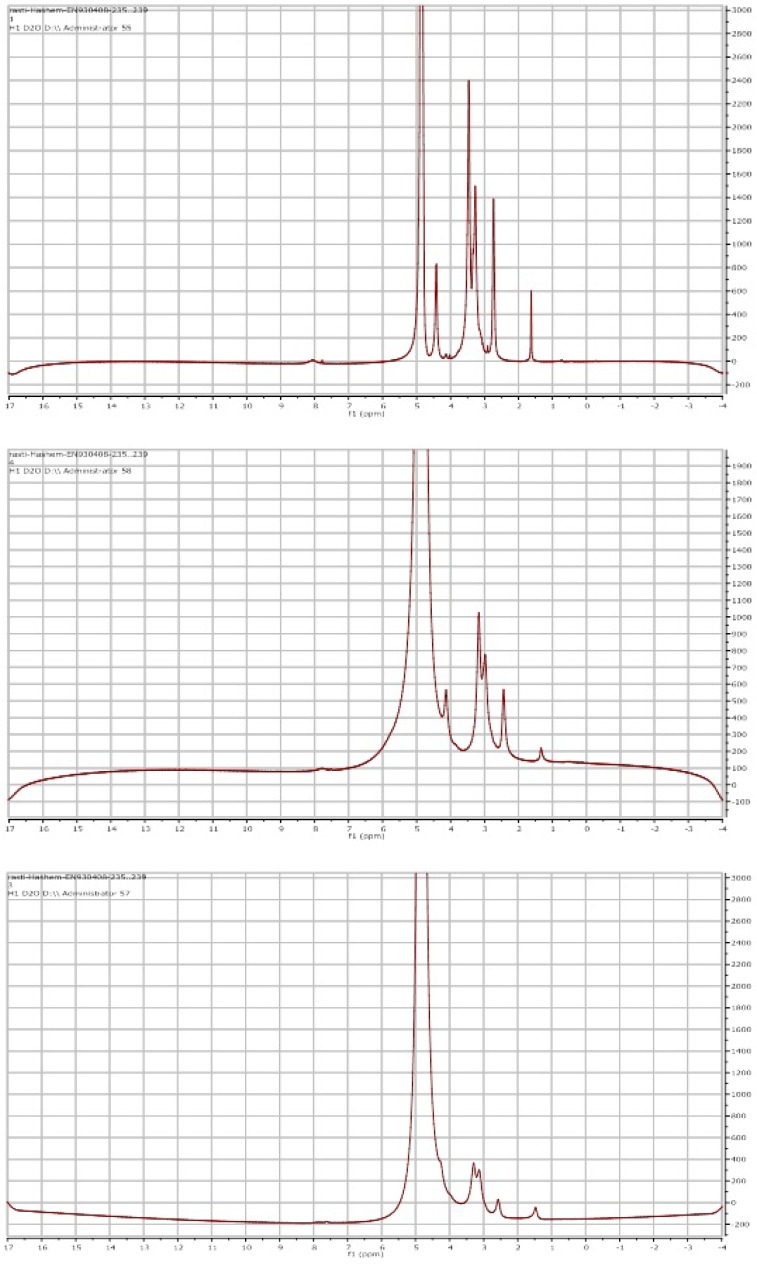
^1^H-NMR spectra (400 MHz) of (A) chitin, (B) chitosan 75% DDA, (C) chitosan 90% DDA

In the ^1^H-NMR spectrum of chitin/chitosan (Figure 4), the signal of methyl protons of acetamide group was appeared at 2.73 ppm. Anomeric proton (C-1 proton) resonance appeared at 4.43 ppm. The other resonances of C2-C6 protons were observed at 3-4 ppm. On the basis of the intensities of the resonances for C-1 proton and methyl protons, one can determine the degree of deacetylation (DDA) from the ^1^H-NMR spectrum as follows:

$\mathrm{DDA}(\%)=\frac{H4.43}{H4.43-H2.73}\times 100$

On the basis of the above formula, the DDA for the chitosan was determined to be 75% and 90% respectively (Figure 4, B&C).

*Scanning electron microscopy (SEM)*

**Figure 5:**
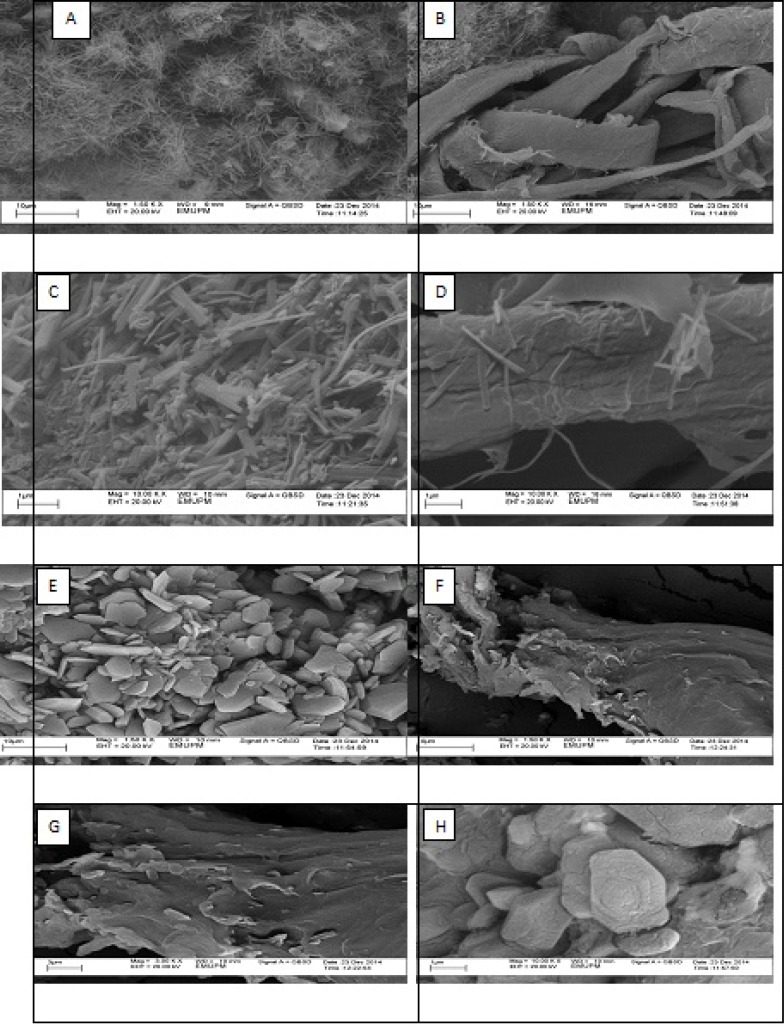
The SEM micrographs for chitin (A, B, C, D) and chitosan (E, F, G, H

The external morphology of chitin and chitosan particles was characterized using Scanning Electron Microscope. Extracted chitin particles were fiber-like and showed distinctly arranged microfibrillar crystalline structure with high diversity and without porosity. The final chitosan demonstrated similar microfibrillar structure with the accumulation of crystalline particles on the fibers in some areas (Figure 5).

*Energy Dispersive X-ray Spectroscopy*

**Figure 6:**
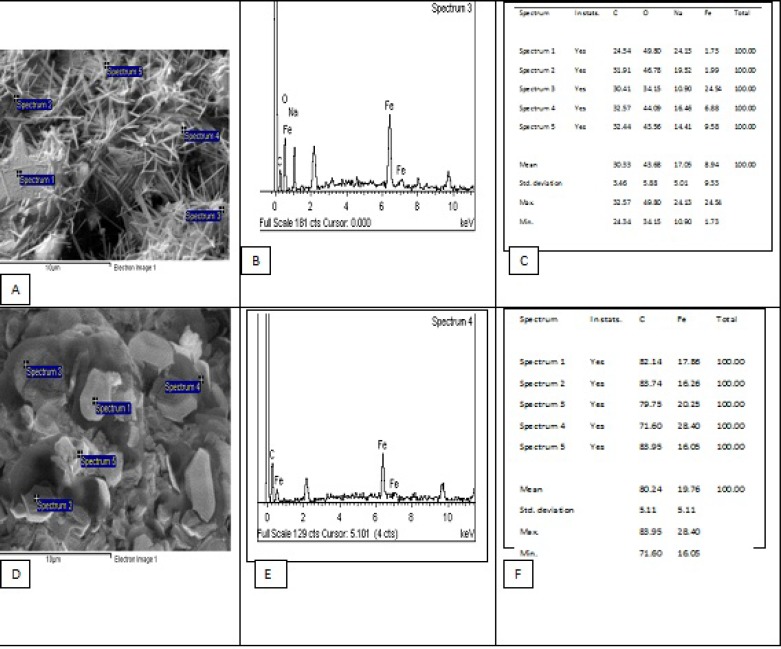
The EDX analysis graph for chitin (A, B, C) and chitosan (D, E, F).

The objective of performing EDX analysis was to investigate the element presence (weight%, Figure 6). The EDX plot of chitin showed the presence of carbon, oxygen, sodium, and iron. Further, EDX plot of chitosan showed the presence of carbon and iron. Therefore, the EDX test demonstrated the presence of iron in the extracted chitin and chitosan. According to EDX results, the average amount of iron in chitin was 8.94 but in chitosan was 19.76.

**Table 1:** Effect of the heating time in oven on DDA% of chitosan

**Row**	**Element**		**Weight (mg)**			**Weight (%)**
Deacetylation by NaOH 45% in 5 hours						
1		Nitrogen		0.137	6.45	
2		Carbon		0.866	40.86	
3		Hydrogen		0.15	7.07	
Deacetylation by NaOH 45% in 15 hours						
1		Nitrogen		0.154	6.51	
2		Carbon		0.917	38.87	
3		Hydrogen		0.149	6.32	
Deacetylation by NaOH 45% in 24 hours						
1		Nitrogen		7.27	7.27	
2		Carbon		38.61	38.61	
3		Hydrogen		6.19	6.19	

**Figure 7:**
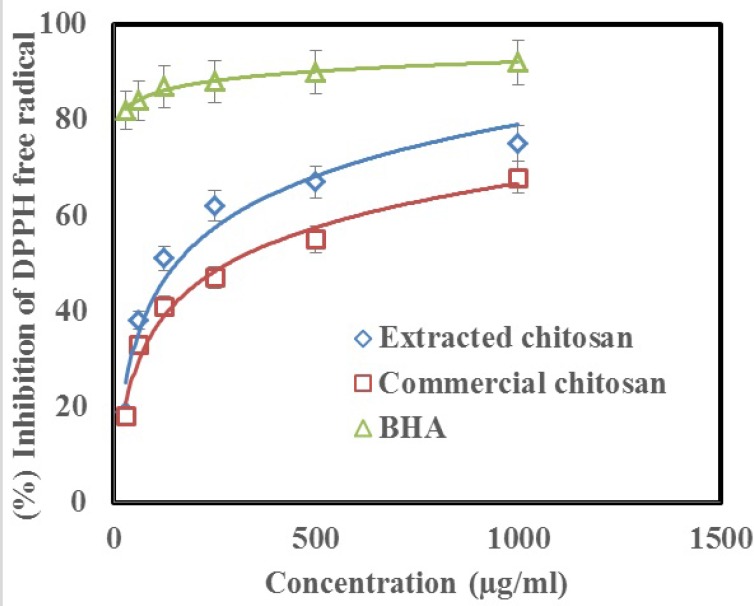
Scavenging activity of extracted and commercial chitosan on DPPH radical when compared with the control (BHA) in similar concentrations

**Figure 8:**
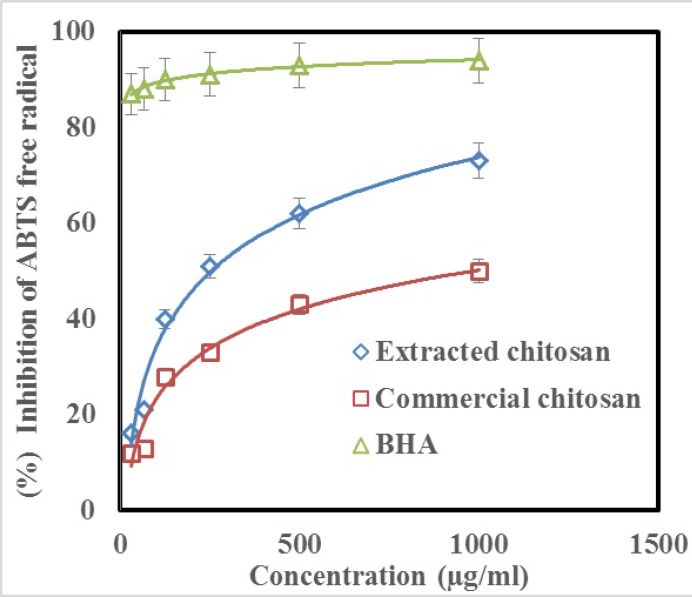
Scavenging activity of extracted and commercial chitosan on ABTS radical when compared with the control (BHA) in similar concentrations

*Antioxidant activity test*

*DPPH radical scavenging activity*

The free radical scavenging activity of chitosan was evaluated by DPPH scavenging. The chitosan demonstrated a dose dependent manner activity. The obtained IC_50_ was 125 µg/mL and 500 µg/mL for extracted and commercial chitosan, respectively. Inhibition of DPPH free radical at the concentration of 1000 µg/mL of extracted chitosan and commercial chitosan was 75% and 68% respectively (Figure 7). Whereas, inhibition of DPPH free radical was 92% at the same concentrations of the standard BHA. 

*ABTS radical scavenging activity*

In order to assay the antioxidant activity of chitosan, ABTS free radical scavenging activity was also measured. Figure 8. shows that the chitosan had an antiradical activity by inhibiting ABTS radical (IC_50_ = 250 μg/mL for extracted chitosan and 1000 μg/mL for commercial chitosan). Further, chitosan showed a dose dependent manner activity similar to which observed in DPPH test. Inhibition of ABTS free radical at the concentration of 1000 µg/mL of extracted chitosan and commercial chitosan was 73% and 50%, respectively (Figure 8). Whereas, inhibition of DPPH free radical was 94% at the same concentrations of the standard BHA. 

## Discussion

 Producing chitosan from the chitin existed through the nature is important; because natural types of chitosan may have novel usages in biological science compared to the available commercial chitosan. Since today, Chitin, as a natural polymer, has been extracted from different natural resources, including crustacean exoskeleton (e.g. shrimps and crabs), insect cuticle, squid pens, and fungi cell membrane. As far as we know, extracting chitin from the Chiton shell is documented for the first time here in this study. Reviewing through the literature showed that the percent of the chitin extracted herein from the Chiton shell (4.3%) is much lower than one reported for the Tiger Prawn (16.75%), Jinga Shrimp (19.13%), Blue Swimming Crab (20.8% for males, and 20.14% for females), Scyllarid Lobster (21.26%), and the Cuttlefish (7.4%) in the Persian Gulf (23). The percent of chitin in the cuttlefish pens, same our finding for the Chiton shell, was reported less than 10% in Kuwait (7.4%) (23) and Egypt (5.4%) (27). As mentioned above, the amount of chitin extracted from the crustaceans in the Persian Gulf (ranged between 16 to 21%) is significantly higher than that reported for the mollusks in the Persian Gulf (7.4% in cuttlefish and 4.3% in Chiton shell). This finding seems natural since the chitin is known as the main substance in the crustaceans’ exoskeleton (23). As previously mentioned, chitin is classified into α-, β-, and γ-types. Abdou *et al* (2008) has been noted that β-chitin has a higher solubility, reactivity, swelling and affinity towards the solvents compared to α-chitin (27). During the experiments, we found high solubility and swelling for the extracted chitin. Therefore, the type of chitin extracted from the Chiton shell may be β-chitin. Based on our results, the concentrations of iron observed in the produced chitosan was 47.2 ppm, which was a very surprising finding as it was about 20 times higher than the concentrations of Fe in the produced chitosan from the crustacean shells in the Persian Gulf (23). The presence of Fe in the Chitons’ radula has been confirmed by several studies (29), which is suggested to be an advantage through the grinding rock to access algae (30). The colour of the produced chitosan here in this study was brownish, which is darker than milky coloured chitosan produced from the crustaceans. Being dark could be a reason of presenting high concentrations of Fe in the Chiton shell. All characterization methods used in this study demonstrated that the products gained after deproteinization and deacetylation were chitin and chitosan, respectively. The results of the characterization tests in this study were compared to those previously reported for crustaceans, because it was the first experience of producing chitosan using the chitin extracted from the Chiton shell. Our results showed that the heating time through the deacetylation has a positive correlation with degree of deacetylation (DDA) (Table 1), which has been noted in other studies (31). Studies show that Fourier transform infrared spectroscopy (FTIR) can be applied for identifying molecules; just like finger print in human (32). 

 FTIR result in our study showed that 1735.26 peak in chitin, marks acetyl group which does not exist in chitosan and this is the successful deacetylation of chitin and finally the preparation of chitosan. Herein we used XRD to detect crystallization, found in the extracted chitin and produced chitosan, and was higher in the chitin (Figure 3). Being crystalline is known as a characteristic of the chitin and chitosan. Further, degree of crystallization is higher in chitin compared to chitosan, since crystallization decreases through the deacetylation (23). Our results have been confirmed by these findings. It should be pointed out that, unlike previous works (27), we dissolved chitin/chitosan in trifluoro acetic acid (TFA). Considering the effects of solvent, temperature and concentration on the chemical shifts of the proton signals of chitin/chitosan, there were slight differences between the chemical shifts of C-1 proton and methyl protons of the acetamide group in our ^1^H-NMR spectrum with those reported previously. According to the obtained results from elemental analysis and ^1^H-NMR, the final degree of deacetylation of chitosan for both methods are very similar. The degree of deacetylation in our study (DDA = 90%) is absolutely similar to the degree of deacetylation from the extracted β chitin from Sepia pharaonis cuttlebone, in Persian Gulf (18). In a previous study, authors extracted chitin from the exoskeleton of the Red Shrimp and monitored the surface of the chitin’s particles using SEM (28). They found that the surface of the particles has a fibril formed without porosity. The same structure has been found in our study (Figure 5). Antioxidant activity is one of the famous functions introduced for chitosan. Several studies have demonstrated that chitosan inhibits the reactive oxygen species (ROS) and barricades the lipid oxidation in the foods and biological systems (33). Some authors suggested that the chitosan produced by the extracted chitin must be treated by the ionizing radiations to show a sufficient antioxidant activity (34). But we gained such this sufficient antioxidant activity without this treatment. The antioxidant activity observed in this study was much higher than that reported for the natural types of chitosan (35). Therefore, the chitosan produced herein may introduce as a natural antioxidant to the pharmaceutical industry. 

## Conclusion

The current study presents the first ever published data of chitin extraction from the Persian Gulf Chiton. Further chitosan was produced herein by the deacetylation of the extracted chitin. The presence of both components was demonstrated and their structure was defined using seven different characterization tests. The produced chitosan contained a significant amount of Fe that was many times higher than that previously reported from the chitosan extracted from the other marine invertebrates. Presence of this amount of Fe could describe that why the produced chitosan was darker compared to the commercial chitosan. Our results showed that the produced chitosan has a stronger antioxidant activity compared to the commercial chitosan, and therefore can be an ideal putative antioxidant source. 

Finally, the chitosan produced here in this study seems very fascinating as an applicant in the pharmaceutical industry.
